# Alcohol Use and Transactional Sex among Women in South Africa: Results from a Nationally Representative Survey

**DOI:** 10.1371/journal.pone.0145326

**Published:** 2015-12-18

**Authors:** Sarah Magni, Nicola Christofides, Saul Johnson, Renay Weiner

**Affiliations:** 1 University of the Witwatersrand, Johannesburg, South Africa; 2 Anansi Health Consulting, Johannesburg, South Africa; 3 Soul City Institute for Health and Development Communication, Johannesburg, South Africa; University of Washington, UNITED STATES

## Abstract

**Background:**

Transactional sex is a risk factor for HIV infection. Alcohol use may increase the risk of transactional sex. No nationally-representative studies have examined the relationship between multiple dimensions of alcohol use and transactional sex in women in South Africa. The aim of the study was to examine the relationship between alcohol dependence, binge drinking and frequency of drinking in the past month and transactional sex in adult women in South Africa.

**Methods:**

A cross-sectional study using multi-stage, cluster sampling collected data from a nationally representative sample of 5,969 women aged 16–55 years in 2012. The analysis conducted for this paper was restricted to women reporting sexual activity in the past 12 months (n = 3,594). Transactional sex was defined as having received money/gifts in exchange for sex with any sex partner in the past year. Alcohol use measures included: alcohol dependence (≥2 positive responses to the CAGE questionnaire); binge drinking (≥4 drinks for women on one occasion); and drinking frequency in the previous month. Logistic regression models were built to test the hypotheses that each dimension of alcohol use was associated with transactional sex.

**Results:**

About 6.3% (n = 225) of sexually active women reported transactional sex. Almost a third (30.6%) of sexually active women had ever drunk alcohol, and 19.2% were current (past month) drinkers. Among lifetime drinkers, 28.0% were alcohol dependent and 56.6% were binge drinkers. Alcohol dependent women were twice as likely to report transactional sex (AOR 2.0, 95% CI 1.1–4.3, p<0.05) than those not alcohol dependent. Binge drinkers were 3.1 times more likely to have had transactional sex (95% CI 1.5–6.6, p<0.01) than non-binge drinkers. There was no significant relationship between frequency of drinking in the past month and transactional sex.

**Conclusion:**

Alcohol dependency and binge drinking are significantly associated with transactional sex in South African women. HIV prevention programmes need to target these women, and address both their alcohol use, as well as the HIV risks associated with transactional sex.

## Introduction

With approximately 23.5 million HIV positive people living in sub-Saharan Africa, the region bears the brunt of the HIV epidemic [1]. South Africa has a very high HIV prevalence with 12.2% of South Africans of all ages living with HIV [2]. Women remain disproportionately affected by HIV. The latest national HIV prevalence survey found that 5.6% of young women aged 15–19 years were infected which was eight times higher than in men (0.7%) in the same age group. In the 20–24 year age range, prevalence was three times higher among women than men: 17.4% compared to 5.1% [2].

One of the factors which increase women’s vulnerability to HIV is engaging in transactional sex [3–5]. For example, Dunkle and colleagues (2004) found, while controlling for the lifetime number of male sex partners and the length of time a woman had been sexually active, transactional sex was associated with HIV seropositivity among women attending antenatal clinics in Soweto, South Africa [3].

Transactional sex is generally defined as a sexual relationship which is primarily motivated by financial or material exchange [6, 7]. However, there is ongoing confusion and disagreement as to what is meant by transactional sex and this concept has been measured and operationalised differently across studies [8]. In transactional sex, cash is most commonly exchanged, but a range of goods or services such as accommodation or transport, may be involved [3, 9, 10]. Although this transaction has both a financial and sexual component, it is differentiated from sex work in that the person who engages in transactional sex does not self-identify as a sex worker, nor is he or she viewed as such by their communities [11]. Research from sub-Saharan Africa has demonstrated that exchange of sex for material or financial resources is commonly practiced and the majority of women who engage in such transactions do not self-identify as sex workers [12–18]. It is worth noting that some studies have conflated transactional sex and sex work [8].

Young women have been reported to engage in transactional sex for a number of reasons including survival, consumption and, in some cases, to increase a sense of agency [9, 18–20]. The transactional nature of these relationships reflects a power imbalance, which for women, often means being less able to influence the timing and nature of sex [14, 17]. Women with little negotiating power to insist on use of condoms experience a higher risk of contracting sexually transmitted infections (STIs), including HIV [19]. Transactional sex is also commonly associated with other risk factors for HIV including intergenerational sex, concurrent sexual partners, unprotected sex [20] and the risk of male-perpetrated intimate partner violence [21].

Another driver of HIV transmission in the sub-Saharan African region is alcohol use, especially more frequent or binge drinking [22–26]. There is a large body of evidence which suggests that alcohol use is strongly associated with HIV incidence [25, 27–30]. Alcohol has been recognised as a key determinant of risky sexual behaviour and as a result, an indirect contributor to the transmission of HIV [31]. A systematic review of the association between HIV infection and alcohol use found that even when all other factors were taken into account, alcohol users had a 57% greater likelihood of being HIV positive than non-drinkers [32]. In recent years, the causal pathway between alcohol intoxication, unsafe sex and HIV acquisition has been more clearly delineated and broadly accepted [33].

Drinking alcohol is associated with a number of risky sexual behaviours including transactional sex [26, 34] and multiple sexual partners [25, 26, 34–36]. Alcohol use, and especially binge (heavy episodic) drinking, has been shown to be associated with unprotected sex and inconsistent condom use [25, 34, 35, 37, 38] with both regular and casual sexual partners [35, 39]. Alcohol use has also been found to be associated with gender-based violence [40], sexual violence [37, 39] and intimate partner violence [41].

Studies examining the relationship between alcohol and risky sexual behaviour have predominately been undertaken in high risk groups such as sex workers [37, 42], bar patrons [42, 43], bar/hotel workers [26] or high risk drinkers [37]. In addition, many of these studies have been venue based. However, as Chersich et al (2007) point out, the relationship between drinking patterns and unsafe sex varies between drinking contexts, population groups and other interacting factors [37]. Findings among high risk groups are likely to differ from the general population [37]. Morojele and colleagues (2006) suggest that a study to look at the extent of risky sexual behaviour among adult and risky drinkers in the general population of South Africa is needed [40].

The quantities of alcohol which people consume and their drinking patterns have an important bearing on their HIV risk. Across a number of studies, a strong dose response relationship between alcohol use and risky sexual behaviour is evident [34]. Frequency of drinking is also of importance. For example, Fritz (2002) found that frequency of drinking in the past week was correlated with the number of episodes of unprotected sex among men [31]. A meta-analysis of African studies found that binge drinking was associated with greater sexual risks than lighter or non-binge drinking [32]. Results from another meta-analysis show that internationally, those who binge drink have double the risk of acquiring HIV compared with non-binge drinkers [27].

South Africa’s National Strategic Plan on HIV, STIs and TB promotes HIV prevention programming for vulnerable populations such as young women who engage in transactional sex and those who abuse alcohol [44]. Understanding the relationship between alcohol use and transactional sex in South African women will assist policymakers and planners to design and implement future interventions to address this important risk factor.

The overall aim of this study was to examine the relationship between alcohol dependence, binge drinking and frequency of drinking in the past month and transactional sex in adult women in South Africa in 2012 among the general population.

## Materials and Methods

We used data collected for the Third National HIV Communication Survey (NCS) [45]. This survey was a nationally representative cross-sectional study of men and women aged 16–55 years conducted between February and May 2012. The analysis conducted for this paper was restricted to female respondents reporting sexual activity in the past 12 months (n = 3,594). Ethical approval for this study was granted by the University of the Witwatersrand Human Research Ethics Committee. Written informed consent was obtained from all participants. Parental assent was also obtained for participants aged 16 and 17 years.

Multi-stage, cluster sampling was used to collect data from a random sample of 10,034 respondents (n = 4,065 men and n = 5,969 women). The sample size was calculated based on the statistical rules applicable for multi-stage probability-based cluster methodologies: the population percentage, the standard error, the desired level of significance and the design effect [45]. Sample weights were introduced to correct for selection bias at the sub-place, household and individual levels. The sample was weighted back to be representative of the population in South Africa in respect of sex, age, race, settlement type and province. Sample weights were benchmarked using the 2007 Community Survey undertaken by Statistics South Africa [45].

Data were collected using a structured, interviewer-administered questionnaire. Fieldworkers used computer assisted personal interviewing to administer the questionnaire, which was translated into all eleven official South African languages. At each selected household, face-to-face interviews were conducted with respondents in their home language by trained interviewers matched as far as possible to the socio-demographics of the respondent.

Respondents were defined as sexually active if they reported having had sex within the past 12 months. Sex was defined in the questionnaire as “when the penis is in the vagina or anus”.

In this study, transactional sex was defined as having received money/gifts in exchange for sex. Receiving money/gifts in exchange for sex was measured by a positive response to a question in the sexual calendar: “In the past year did you receive gifts or money from this person in order to have sex with him/her?”. Transactional sex was measured across all types of sexual partnerships reported in a sexual calendar.

*Lifetime drinking* was measured by asking respondents “Have you ever had an alcoholic drink?”. Current drinking was measured by an item that asked “During the past month, how many times did you have an alcoholic drink?”. Those who responded that they had had one or more drinks were categorised as “Drank any alcohol in the past month”, and those who reported that they had not had any alcoholic drinking the past month were coded as “Did not drink any alcohol in the past month”. This is consistent with the way current drinking has been measured elsewhere [46].

*Alcohol dependence* was assessed using the four-item CAGE questionnaire [47]. The specific items, with yes/no responses, were: “Have you ever felt you should cut down on your drinking?”; “Have people annoyed you by criticising your drinking”; “Have you ever felt bad or guilty about your drinking?”; and “Have you ever had a drink first thing in the morning to steady your nerves or get rid of a hangover (eye-opener)?”. Claasen (1999) reports that the CAGE questionnaire showed a sensitivity of 100% and a specificity of 78% for alcohol dependence in rural South Africa [48]. As recommended by Dhalla and Kopec (2007)[49] and in line with other studies in South Africa, respondents were classified as alcohol dependent if they responded “yes” to at least two items [46, 47].There was acceptable internal consistency with this measure (Chronbach alpha = 0.7).

*Binge drinking* was measured using an item that asked: “How often do you have four or more drinks on one occasion?” [50]. Women who responded that they had done so “Less than once a month”; “A few times a month”; “Almost every week; and “Almost every day” were categorised as “Binge drinkers”. “Non-binge drinkers” were those who answered “Never” or “Hardly ever”.

*Frequency of drinking* was measured using the question: “During the past month, how many times did you have an alcoholic drink?”. Response options included: “Almost every day”; “Several times per week”; “At least once a week”‘ “At least once a month”; and “Never”.

Ideation refers to new ways of thinking that diffuse within a culture by means of social interaction [51]. It encompasses cognitive, emotional, and social determinants of behaviour such as knowledge, beliefs, perceived risk, self-efficacy, personal advocacy and social norms [52]. The ideational factor of relevance to this paper is HIV prevention knowledge. HIV prevention knowledge was measured using a scale of ten items which measured knowledge of HIV prevention methods.

HIV status was measured through self-report. Individuals who reported ever testing for HIV were asked if they knew their HIV status. Those who answered in the affirmative, and who were comfortable sharing their status, were asked what their status was.

Violence was measured as having been in a physical fight in the past 12 months.

### Statistical Analysis

Data analysis was undertaken in Stata (version 12.0, STATA Corp,. College Station, Texas, USA). All analysis took into account the multistage, cluster sample design of the study.

To test the hypothesis that multiple dimensions of alcohol use increased transactional sex among women, logistic regression models were built that modelled alcohol dependence, binge drinking and frequency of drinking in the past month and transactional sex. Alcohol variables were only added into the models where they were significant (p<0.1) in the univariate analysis.

Analyses controlled for other covariates which were selected on a basis of theoretical relevance and being independently associated with the outcomes in bivariate analysis (*p-*value <0.10). Covariates included ideational factors, exposure to HIV communication programmes and behaviour. e.g. violence.

These models were developed using a backwards stepwise logistic approach. All significant variables were entered into the model, and at each step the least significant variable was removed until all the remaining variables had a statistically significant contribution to the model or were theoretically important (based on the literature). Variables were eliminated based on p-values > 0.1. Age and cohabitation status were controlled for in each model.

The results of the final logistic regression models were reported using Adjusted Odds Ratios (AORs) and 95% confidence intervals (CIs).

## Results

This study found that 86.3% (n = 4,978) of women had ever had sex and 74.7% (n = 3,594) of these reported having sex in the 12 months prior to the survey. The socio-demographic characteristics of women reporting sexual activity in the past 12 months (n = 3,594) who participated in the study are presented in Table 1. The median age of women reporting sexual activity in the past 12 months was 29 years and the interquartile range was 14 years. Some 21.5% of respondents were aged 20–24 years and one in five (20.2%) were 25–29 years of age. The majority of respondents (78.1%) were Black African. Over a third had completed high school while 39.8% had attended some high school. Nearly six out of ten (59.5%) women were unemployed, 8.5% were students, while two-thirds (64.1%) lived in urban areas. Some 13.1% of women reporting sexual activity in the past 12 months and who had ever tested for HIV and knew their status reported being HIV positive.

**Table 1:** Socio-demographic characteristics of women 16–55 years reporting sexual activity in the past 12 months.

Characteristics	Number	Unweighted percentage (%)	Weighted percentage (%)
**Age (years) *n = 3*,*594***			
16–19	247	6.9	6.4
20–24	784	21.8	21.5
25–29	768	21.4	20.2
30–34	603	16.8	16.5
35–39	431	12.0	12.9
40–44	308	8.6	9.3
45–49	229	6.4	6.3
50–55	224	6.2	7.0
*Median (IQR)*	*29 (14)*		
**Race *n = 3*,*593***			
Black	2,975	82.8	78.1
Coloured	525	14.6	8.5
Indian	28	0.8	2.5
White	65	1.8	10.9
**Education *n = 3*,*588***			
No schooling	37	1.0	1.3
Up to primary school	324	9.0	9.0
Up to grade 11	1,545	43.1	39.8
Matric	1,270	35.4	34.9
Tertiary	412	11.5	15.1
**Employment *n = 3*,*543***			
Employed	1,055	29.8	32.1
Unemployed	2,198	62.0	59.5
Student	290	8.2	8.5
**Settlement type *n = 3*,*594***			
Urban	2,468	68.7	64.1
Rural	1,126	31.3	35.9
**Self-reported HIV status *n = 2*,*471***			
HIV positive	316	12.8	13.1
HIV negative	2,115	87.2	87.0

### Transactional sex

About 6.3% (n = 225) of women reporting sexual activity in the past 12 months reported receiving money/gifts in exchange for sex with any of their sex partners in the past 12 months. Using weighted data, this translates into around 560,000 women in South Africa reporting transactional sex in the previous year.

Table 2 shows that knowing one’s HIV status was significantly associated with receiving money/gifts in exchange for sex among women. Women who knew their HIV status were significantly less likely (p = 0.02) to receive money/gifts in exchange for sex (86.0% vs 93.8%). There was a borderline significant relationship between self-reported HIV positive status and engaging in transactional sex (p = 0.06). Women who were in a physical fight in the past 12 months were significantly more likely (p<0.001) to have engaged in transactional sex in exchange for money/gifts than those who did not (14.6% vs 6.9%).

**Table 2:** Associations between socio-demographic characteristics, ideational factors, behaviour, and transactional sex among women 16–55 years reporting sexual activity in the past 12 months n (%).

	Received money/gifts in exchange for sex with any sex partner in the last 12 months *n = 3*,*574*		
	Yes n (%)	No n (%)	P value
**Socio-demographic characteristics**			
***Age***			
16–24	77 (29.7)	949 (27.9)	0.1
25–34	97 (43.9)	1,263 (36.1)	
35–55	51 (26.4)	1,137 (36.1)	
***Marital status***			
Single/ Divorced/Widowed	102 (41.2)	995 (29.6)	0.1
Not married or living together but in a steady relationship	44 (19.4)	874 (23.8)	
Not married, but living with sexual partner	31 (15.7)	478 (14.8)	
Married, living together	43 (21.7)	876 (27.3)	
Married not living together	5 (2.1)	126 (4.5)	
***Education***			
No/ primary school	18 (7.0)	342 (10.5)	0.5
Up to grade 11	98 (37.6)	1,438 (39.8)	
Matric	83 (38.1)	1,178 (34.7)	
Tertiary	25 (17.3)	386 (15.0)	
***Employment***			
Unemployed	145 (61.5)	2,042 (59.3)	0.7
Employed	59 (31.6)	988 (32.1)	
Student	17 (6.9)	273 (8.6)	
***Food security***			
Food insecure	60 (22.5)	547(16.9)	0.1
***Settlement type***			
Urban	117 (60.4)	2,338 (64.4)	0.5
Rural	108 (39.6)	1,011 (35.6)	
**Ideational factors**			
***Knowledge of HIV prevention methods***			
No/low knowledge	42 (19.0)	750 (22.3)	0.1
Medium knowledge	73 (32.2)	1,414 (40.6)	
High knowledge	110 (48.8)	1,185 (37.1)	
**HIV status & exposure to HIV communication**			
***Knows HIV status***			
Yes	156 (86.0)	2,641 (93.8)	0.0
***Self-reported HIV status***			
HIV positive	27 (19.1)	289 (13.2)	0.1
***HIV communication programmes***			
Exposed to at least one HCP	202 (89.5)	2,927 (84.4)	0.1
**Behaviour**			
***Violence***			
Been in a physical fight in the past year	36 (14.6)	239 (6.9)	<0.001
**Alcohol use**			
***Binge drinking***			
Yes	52 (78.5)	20 (55.0)	<0.001
***Frequency of drinking***			
Never	17 (21.0)	361 (35.8)	0.0
At least once a month	22 (33.5)	299 (30.8)	
At least once a week	19 (23.1)	187 (23.1)	
Several times per week	7 (14.5)	89 (8.8)	
Almost every day	8 (7.9)	18 (1.6)	
***Alcohol dependence***			
Yes	37 (42.0)	307 (26.9)	0.0

### Alcohol use

Nearly a third (30.6%) of women reporting sexual activity in the past 12 months (n = 1,048) reported ever having had an alcoholic drink and 19.2% (n = 650) were current (past month) drinkers. Rates of lifetime drinking differed substantially across the country. Only 15.9% of women who were sexually active in the past 12 months in Limpopo Province reported lifetime drinking compared with 84.2% in Northern Cape Province.

Of those who reported ever drinking alcohol, 28.0% (345) were classified as alcohol dependent using the CAGE questionnaire. More than half, 56.6% of women (n = 535) who had ever drunk alcohol, were classified as binge drinkers. Using weighted data, this equates to an estimated 1.5 million women in South Africa.

### The relationship between alcohol and transactional sex with any sex partner in the past 12 months

In the multivariate regression model, adjusting for confounding variables, women classified as binge drinkers were three times more likely to have engaged in transactional sex (AOR 3.1, 95% CI 1.5–6.6) than women who had ever had an alcoholic drink but who did not report binge drinking. This is shown in Table 3. Women who were alcohol dependent were 2.2 times more likely to report transactional sex (AOR 2.2, 95% CI 1.1–4.3). There was no significant relationship between frequency of drinking in the past month and transactional sex.

**Table 3:** Multivariate logistic regression results for transactional sex with any sex partner in the past 12 months and binge drinking, frequency of drinking in the past month and alcohol dependence among women^*^.

	Crude odds ratio	Adjusted odds ratio	95% CI	P value
**Binge drinking**				
No	Ref	Ref		
Yes	2.5	3.1	1.5–6.6	<0.01
**Frequency of drinking in the past month**				
Never	Ref	Ref		
At least once a month	1.6	1.5	0.6–3.3	0.4
At least once a week	2.2	1.4	0.6–3.5	0.5
Several times per week	1.7	2.2	0.7–6.9	0.2
Almost every day	9.4	3.7	0.6–22.6	0.2
**Alcohol dependence**				
No	Ref	Ref		
Yes	2.0	2.2	1.1–4.3	0.0

*Controlled for age; cohabitation status; settlement type; food security; involvement in physical violence; HIV prevention knowledge; and HIV status.

## Discussion

**Fig 1:**
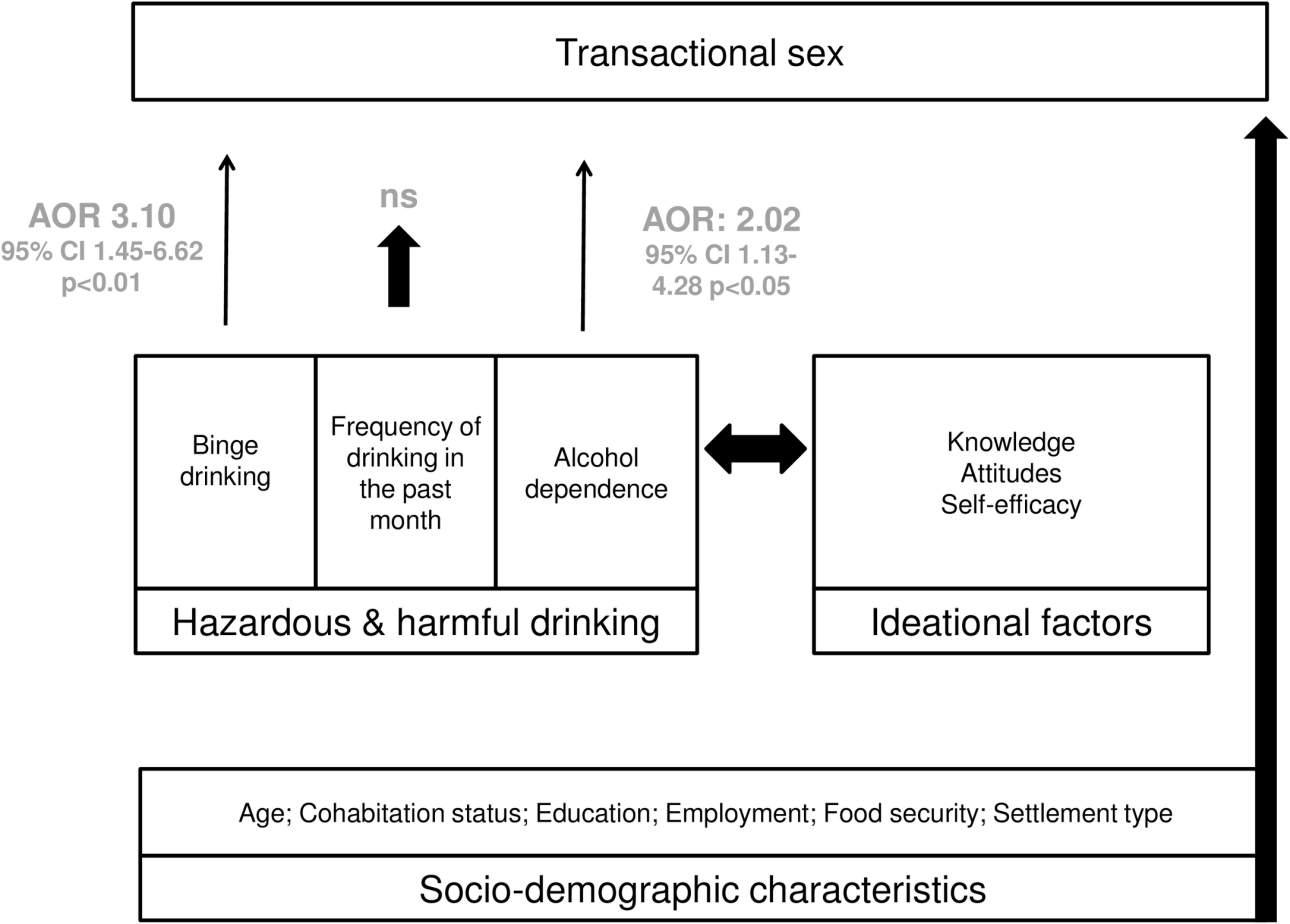
The relationship between hazardous drinking and transactional sex based on the results of this study.

This study aimed to test the hypotheses that multiple dimensions of alcohol use were associated with transactional sex among women in the South African general population. Results support the hypotheses that binge drinking and alcohol dependence increases the likelihood of engaging in transactional sex. A conceptual model describing the relationship between alcohol use and transactional sex has been proposed (Fig 1). According to this model, socio-demographic characteristics have an independent effect on transactional sex. The model proposes that there is a bi-directional relationship between hazardous drinking and ideational factors.

The relationship between alcohol dependence and transactional sex among women is worth further consideration. In a study undertaken with patrons of alcohol-serving establishments in Cape Town, Pitpitan and colleagues (2013) found that women who traded sex were more likely to be alcohol dependent [43]. In Pitpitan et al’s study (2013) the term problem drinking was used to characterise those who answered “yes” to at least two items of the CAGE questionnaire. This is the same measure used in this study, where the term alcohol dependence is used to describe individuals with a CAGE score of two or more. Viewed together, these findings suggest that the relationship between alcohol dependence and transactional sex may be bi-directional, which is what Townsend and colleagues propose in their model outlining how alcohol use and transactional sex are linked to risky sexual behaviour [53]. We have not found any evidence to support the idea that the relationship between binge drinking and transactional sex is bi-directional.

This study also found that 6.3% of women reporting sexual activity in the past 12 months reported that they had recently engaged in transactional sex. This is consistent with findings from 12 sub-Saharan African countries where the prevalence of receiving money or gifts among women aged 15 years and older ranged from 1.8% to 11% [6]. However, this rate is substantially lower than that found among women attending antenatal clinics in Soweto (21.1%) [3]. This discrepancy may be because the NCS was a national survey whereas Dunkle and colleagues’ study (2004) was only conducted in one province and in view of the different age bands-the national study included women up to the age of 55 years who are arguably less likely to engage in transactional sex.

Study results support the assertion that while levels of alcohol use in South Africa are relatively low, levels of hazardous and harmful drinking are high [46, 54].This study found that 28% of female drinkers were alcohol dependent, which is higher than results from the South African Demographic and Health Survey of 1998 (10%) and of 2003 (6.9%) [55, 56]. Of note is that over half of sexually active female drinkers, equating to roughly 1.5 million women, were classified as binge drinkers. This is consistent with other studies which found that although a relatively low percentage of women drink, those who do drink, do so at high risk levels [57].

These high levels of binge drinking among women may be partly explained by the growing body of research which shows that female drinking is often related to defiance of gender norms [58] and an increased sense of agency [59]. A number of studies have described the socially-scripted phenomenon which occurs in taverns. It is common place for men to buy women drinks and it is mutually understood that this is likely to end in sex [53, 59, 60]. Qualitative studies have revealed that alcohol is a common and desired form of currency in transactional relationships [41, 43]. Some women demonstrated increased agency by participating in the transactional sex dynamic [18, 59].

Another potential reason why hazardous alcohol use is high may be the lack of awareness around safe drinking relative to non-drinking. In neighbouring Namibia, LeBeau and Yoder (2009) found that the concept of moderate drinking was not one which was commonly understood, with few people understanding the difference between non-drinking and harmful drinking [60] This may well be the case in South Africa too. The high frequency of heavy drinking amongst women is likely to be further promoted by aggressive marketing of alcohol increasingly targeted at young women [61].

The results of this study should be interpreted in light of its limitations. Firstly, the NCS was cross sectional, limiting an assessment of causality conclusions between alcohol and risky sexual behaviour because measurement of exposure and effect occur simultaneously [62]. Secondly, there were potentially issues of measurement bias in relation to exposure. Alcohol measures are especially subject to recall bias and underestimation [37, 63]. The NCS questionnaire asked respondents who drank: “How often do you have (for men) five or more and (for women) four or more drinks on one occasion?”. This question did not include a timeframe [50], which is necessary to quantify frequency of binge drinking “to differentiate “binge drinking” from “alcoholism” or “alcohol dependence”“[64]. In addition, this study did not make use of AUDIT which has been recommended for alcohol measurement [65]. Not only does this make it difficult to compare to other studies in South Africa but literature suggests that structured instruments, like AUDIT, generally perform better than quantity-frequency questions [66].

Finally, there may have been issues in relation to measurement bias for the outcomes. Under-reporting of risky sexual behaviours is common [67] and this may have led to an underestimate of transactional sex. It is also likely that measurement of transactional sex in the NCS was incomplete. A more complete definition would have included alcohol and/or drugs as well as gifts and/or money as in Pitpitan and colleagues’ study (2014) where participants were asked: “Has someone given you money, alcohol, drugs or a place to stay in exchange for sex in the past 4 months?” [43].

Notwithstanding the above limitations, the findings of this study are important. This is the first national published study we are aware of which examines the relationship between multiple and more nuanced measures of alcohol use—alcohol dependence, binge drinking and frequency of drinking in the past month—and transactional sex in women. Although levels of lifetime drinking among women are quite low, the levels of binge drinking and alcohol dependency are scarily high. Findings demonstrate a clear, significant association between both binge drinking and alcohol dependence, and transactional sex amongst South African women in the general population. This confirms that alcohol misuse is an important driver of HIV risk among South African women, and tackling alcohol needs to be central to HIV prevention efforts especially for young women who are particularly vulnerable to transactional sex. Modelling data from Kenya suggests that with widespread uptake of interventions targeting unhealthy alcohol use, HIV infections could be reduced by five percent and some 18,000 AIDS-related deaths could be averted [68]. Moreover, studies have concluded in countries with severe HIV epidemics that designing and implementing interventions to promote safer drinking practices together with interventions to reduce risky sexual behaviour may have the potential to reduce HIV transmission. These data are relevant to South Africa where unsafe drinking and risky sex are both prevalent in the context of a generalised epidemic [68, 69].

The findings of this study have several implications for HIV prevention policy. Since young women and girls are particularly vulnerable to HIV, it is clear that programmes which address both alcohol and HIV prevention are needed for this group. This is especially important in light of the fact that alcohol consumption in sub-Saharan Africa is expected to increase in the next ten years due to improving economic conditions and aggressive alcohol marketing [70]. Programming needs to include interventions to prevent alcohol misuse particularly among young women as well as specifically targeting women who are already alcohol dependent and binge drinkers.

Further intervention research to design and evaluate the impact of such interventions on transactional sex and HIV incidence, within the South African context, would be helpful to inform programme implementation. While the need for a randomised controlled trial that evaluates the effectiveness of an alcohol focused intervention on HIV incidence has been identified, it does not focus specifically on women as a key population and/or include transactional sex as an outcome [71].

There are a number of other research implications arising from this study. It would be helpful to use other national level data, such as from the HIV prevalence surveys [2], to further analyse the relationship between different measures of alcohol use, transactional sex and HIV longitudinally. Longitudinal studies could be undertaken in the future to explore the hypothesised pathways between alcohol and transactional sex and HIV in relation to other potential paths linked to transactional sex such as experiences of intimate partner violence.

It is clear that national level programmes are needed as this study shows that both drinking and transactional sex is found in adult women throughout the country. Such programmes need to consider the multiple levels of intervention including increasing risk perception of hazardous drinking particularly for women; shifting drinking norms including those that facilitate transactional sex in taverns, creating safer drinking environments and, limiting/banning alcohol marketing towards women.
